# Motor performance and higher associative cortical networks in adolescents with neonatal hypoxic‐ischaemic encephalopathy treated with therapeutic hypothermia

**DOI:** 10.1111/dmcn.16371

**Published:** 2025-06-22

**Authors:** Gustaf Håkansson, Mimmi Eriksson Westblad, Maria Örtqvist, Ulrika Ådén, Mats Blennow, Peter Fransson

**Affiliations:** ^1^ Department of Pediatrics Karolinska University Hospital Stockholm Sweden; ^2^ Department of Clinical Science, Intervention and Technology Karolinska Institutet Stockholm Sweden; ^3^ Medical Unit Allied Health Professionals Karolinska University Hospital Stockholm Sweden; ^4^ Department of Women's and Children's health Karolinska Institutet Stockholm Sweden; ^5^ Department of Biomedical and Clinical Sciences Linköping University Linköping Sweden; ^6^ Department of Clinical Neuroscience Karolinska Institutet Stockholm Sweden

## Abstract

**Aim:**

To investigate the interaction between specialized motor networks and higher associative brain networks for motor performance in adolescents exposed to neonatal hypoxic‐ischaemic encephalopathy (HIE).

**Method:**

In this prospective, population‐based cohort study of children (*n* = 66) with neonatal HIE treated with therapeutic hypothermia, follow‐up was performed at age 10 to 12 years with resting‐state functional magnetic resonance imaging and assessment of motor performance with the Movement Assessment Battery for Children, Second Edition (MABC‐2). Brain–behaviour analysis was performed using statistical enrichment analysis and was compared to a control group (*n* = 43) using the McNemar test.

**Results:**

The final analysis included 35 children in the cohort with HIE (mean [SD] age at magnetic resonance imaging = 11 years 2 months [9 months]) and 21 children in the control cohort (10 years 2 months [8 months]). Motor performance (assessed with the MABC‐2 total score and all subdomains) was reduced in the cohort with HIE compared to the controls and was significantly associated with several clusters of brain network connections. A significant group difference was found in the MABC‐2 aiming and catching subdomain, which correlated with clusters of functional connectivity between the somatomotor and default mode networks in the cohort with HIE.

**Interpretation:**

Motor impairment after therapeutic hypothermia‐treated neonatal HIE is connected to alternative neural processing between motor and cognitive networks.

## Graphical abstract

Several functional connections between ROIs in the somatomotor (purple) and the default mode network (gray) were significantly associated with the MABC‐2 “aiming and catching” sub‐domain in the HIE cohort in comparison to controls. The colors of the connections represents the correlation coefficient with either negative (light blue to magenta) or positive (green to red) slope.

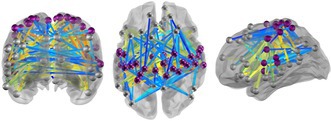

**This original article is commented on by Galanides et al. on pages** 11–12 **of this issue.**

**Abbreviations**
BOLD: blood oxygen level‐dependent
DCD: developmental coordination disorder
DMN: default mode network
fMRI: functional magnetic resonance imaging
HIE: hypoxic‐ischaemic encephalopathy
MABC‐2: Movement Assessment Battery for Children, Second Edition

**What this paper adds**

- Children exposed to hypoxic‐ischaemic encephalopathy (HIE) depend on higher associative brain networks for motor performance.
- Somatomotor‐related network alterations after HIE are similar to developmental coordination disorder.
- Impaired motor function after HIE could depend on specific cognitive functions.

Motor impairment is common after neonatal hypoxic‐ischaemic encephalopathy (HIE), even in the absence of cerebral palsy (CP) and treatment with therapeutic hypothermia.^1^, ^2^, ^3^ In children exposed to HIE, lower scores obtained in the assessment of motor skills with the Movement Assessment Battery for Children, Second Edition (MABC‐2) has been associated with decreased cognitive performance, such as inattention.^4^ Motor impairment can appear even after school age, at a time when many higher cognitive functions mature,^5^ thus further supporting the hypothesis of a cognitive contribution to these symptoms. With functional magnetic resonance imaging (fMRI), the absolute level of deoxygenated haemoglobin is measured over time. This blood oxygen level‐dependent (BOLD) signal is driven by regional changes in blood oxygen concentration and blood flow that in turn are connected to neural activity in a process called neurovascular coupling. Therefore, it can be used as an indirect measure of brain activity.^6^ The BOLD signal continuously changes across the brain, reflecting spontaneously ongoing low‐frequency activity. Consequently, when an individual is scanned over time without specific tasks (i.e. at resting state), functional connectivity across all brain regions can be estimated by computing the correlation of simultaneous brain activity.^7^ Spontaneously connected brain regions constitute networks (i.e. resting‐state functional connectivity networks) that show intraindividual and interindividual stability and have been attributed to specific brain functions, including motor function and specific cognitive functions.^8^ At the time of neonatal HIE, the different functional networks of the newborn infant brain have reached different stages of development.^9^ The somatomotor network develops early and already has an adult‐like spatial architecture at birth, while higher‐order associative networks develop later in childhood and adolescence.^9^, ^10^, ^11^ In a recent study, we assessed whole‐brain functional connectivity in a cohort of children exposed to therapeutic hypothermia‐treated neonatal HIE but failed to detect any group‐level significant abnormalities of the somatomotor network compared with typically developing controls.^12^ In this study, we aimed to investigate if altered interaction between functional connectivity networks with or without specific motor function was related to motor performance in this cohort of young adolescents exposed to therapeutic hypothermia‐treated neonatal HIE.

## METHOD

### Participants

This study is a follow‐up of a population‐based cohort (*n* = 66), who were recruited neonatally in Stockholm, Sweden between January 2007 and December 2009; it included all children meeting the criteria for therapeutic hypothermia treatment of neonatal HIE. All 52 eligible children in the cohort with HIE (eight children deceased, one child excluded because of Prader–Willi syndrome, and five children lost to follow‐up because of moving abroad) were invited for assessment at age 10 to 12 years. Information regarding the demographic and neonatal characteristics, as well as clinical outcome, motor development, and whole‐brain functional connectivity has been presented previously.^5^, ^12^, ^13^ A control group of 43 children, born in Stockholm between 36 weeks and 42 weeks of gestation from singleton pregnancy, and with a 5‐minute Apgar score greater than 3, was recruited separately through random identification from the Swedish Medical Birth Registry and participated in the same data collection, with assessment of fMRI data at age 10 years and MABC‐2 at age 12 years. The study was approved by the Ethical Review Board in Stockholm and conformed to the Declaration of Helsinki (2009/735–31/4, 2010/850–31/1, 17 2012/617–32, 2016/1921–32, 2019–01447, 2020–03318). Written informed consent was obtained from all caregivers; children gave assent to participate.

### Assessment of motor performance

Motor performance was assessed with the standardized MABC‐2, which has shown good validity and measure characteristics for motor competence in this age group.^14^ The cohort with HIE and the control cohort were assessed by an experienced paediatric physiotherapist who was not blinded to group allocation. The MABC‐2 consists of eight items divided into three subdomains—manual dexterity, aiming and catching, and balance—which can be further combined into a total score. Thus, for each participant, four standardized motor performance parameters were computed and subsequently used for the brain functional connectivity analysis.

### Functional magnetic resonance image acquisition

The fMRI was performed using a Sigma 3 Tesla (GE HealthCare, Milwaukee, Wisconsin, USA) magnetic resonance scanner at the MR Research Center, Karolinska Institutet, Sweden. T1‐weighted, two‐dimensional spin echo images (repetition time = 6.2, echo time = 2.7, inversion time = 400 ms, flip angle 12 degrees, acquisition matrix 256 × 256 × 160 pixels, voxel size 1.0 × 0.94 × 0.94 mm^3^) were obtained in the axial plane using a 64‐channel head coil. Resting‐state fMRI data (T2‐weighted echo planar imaging) were acquired during a 10‐minute scan (300 whole‐brain image volumes, repetition time/echo time 2000s/30ms, flip angle 70 degrees, 3 × 3 × 3 mm voxel size). All participants were instructed to lie still and not fall asleep during resting‐state image acquisition; no sedation was used.

### Image preprocessing

Preprocessing of both anatomical and functional imaging data was performed using fMRIPrep v20.2.4 (https://fmriprep.org/en/stable/)^15^ and mainly followed the same procedure as described in Håkansson et al.^12^ Non‐uniform signal intensity in T1‐weighted images in native space was corrected with N4BiasFieldCorrection^16^ before skull‐stripping with antsBrainExtraction and volume‐based spatial normalization to a standard space template (MNI152NLin2009cAsym)^17^ through non‐linear registration with antsRegistration 2.3.3.^18^

The quality of the fMRI data was assessed through visual inspection and using quantitative metrics extracted with the fMRI Quality Control tool v0.16.1 before further processing.^19^ Because of a general tendency of increased head movements towards the end of the scan, the last minute (30 volumes) was excluded for all participants, resulting in 9 minutes (270 volumes) of resting‐state BOLD data for the functional connectivity analysis. A functional reference volume was created in fMRIPrep using the median of a motion‐corrected subset of the BOLD volumes for further co‐registration with FLIRT (FSL v5.0.9)^20^ to the T1‐weighted volume using the boundary‐based registration cost function with six degrees of freedom. Movement regressors (i.e. three translational and three rotational) were extracted using McFLIRT^21^ (FSL v5.0.9) and used for resampling of the functional images into native space using a rigid head movement model. Finally, resampled functional volumes were slice‐time‐corrected to 0.976 s in 3dTshift (Analysis of Functional NeuroImages)^22^ and subsequently normalized to the MNI brain atlas space.

### Denoising of functional magnetic resonance imaging data

BOLD signal intensity time series were extracted from the preprocessed functional images by combining the 7‐network, 200‐node Schaefer parcellation,^23^, ^24^ King parcellation of the cerebellum,^25^ and Oxford Harvard subcortical parcellation.^26^, ^27^, ^28^, ^29^ In total, our choice of brain parcellation produced BOLD signal time courses for 223 brain regions divided into nine networks (visual, somatomotor, dorsal attention, ventral attention/saliency, limbic, control, default mode, cerebellar, and subcortical). Signal denoising regression was performed for the following 36 confounding parameters (six for each category):^30^ head‐motion parameters; average signal from the white matter; cerebrospinal fluid; global signal; and their first and second derivatives. After BOLD signal regression denoising, participant head movement data censoring was performed by removing all volumes exceeding a threshold of framewise displacement of 0.3 mm (framewise displacement),^31^, ^32^ along with the two previous and two subsequent volumes.^33^ If the number of retained image volumes were fewer than 120, the corresponding data set was excluded from further analysis. Data were temporally bandpass‐filtered (0.008–0.1 Hz); however, no spatial smoothing was applied because the mean time series were extracted using a parcellation scheme. All denoising procedures were carried out in Python v3.9 using Nilearn v0.10.0 (https://nilearn.github.io/stable/index.html).^34^

### Statistical analysis

#### Preliminary analysis

Cohort differences were assessed with regard to sex, gestational age, birthweight, age at magnetic resonance scanning, and age at MABC‐2 assessment using a *t*‐test for continuous data and a *χ* ^2^ test for categorical data. Raw scores from each of the three MABC‐2 subdomains and the total score were converted to a standard score with regard to normative data (mean = 10, SD = 3). The distributions of the MABC‐2 standardized test results were checked for normality using the Shapiro–Wilk test to determine the appropriate statistical method for the brain–behaviour analysis (i.e. Pearson's rank correlation coefficients for normally distributed MABC‐2 scores and Spearman's rank correlation coefficients for a non‐normal distribution). In addition, the group differences (cohort with HIE and control cohort) of the MABC‐2 scores were assessed using a two‐tailed independent *t*‐test for normally distributed data and the Mann–Whitney *U* test for non‐normally distributed data, correcting the *p*‐value for the multiple comparison of all four MABC‐2 scores using Bonferroni correction.

#### Brain–behaviour analysis

For the brain–behaviour association analyses, we used a network implementation of the enrichment analysis, which originally stemmed from genome‐wide association studies.^35^ Briefly, the enrichment analysis used in this study (Network Level Analysis Toolbox v1.0)^36^ in MATLAB (release R2022a, MathWorks, Natick, MA, USA) uses a model‐based data reduction approach to extract statistically significant network features with a data‐driven, permutation‐based false positive rate procedure to account for multiple comparisons. Enrichment analysis has been used previously in several studies where the relationship between brain connectivity and behaviour was studied.^36^, ^37^, ^38^, ^39^, ^40^

Connectivity matrices (Pearson's rank correlation coefficients) were computed from the denoised and parcellated BOLD signal time series for each participant. After Fisher z‐score transformation, all pairwise brain region functional connectivity measures were correlated with each MABC‐2 measure using Pearson's rank correlation coefficients for scores with a normal distribution and Spearman's rank correlation coefficients for non‐normally distributed scores, thus creating eight (four motor performance parameters for both the control group and the group with HIE) separate brain–behaviour correlation matrices. Statistical significance for the enhancement of strong correlation (*p* < 0.05) between pairwise brain functional connectivity and motor performance was tested at the network level with an enrichment methodology, using two tests for the assessment of each functional network pair; a one‐degree‐of‐freedom *χ* ^2^ test was used to determine the difference between observed and expected strong brain–behaviour correlations, while a hypergeometric (Fisher's exact) test was used to investigate the likelihood of observing a strong correlation. Differences in brain functional connectivity associated with motor performance between the cohort with HIE and the control cohort were subsequently tested at the network level using the McNemar *χ* ^2^ test for all network pairs that were significantly enriched at the previous step. Statistical significance was assessed using permutation (10 000 permutations) to generate a null distribution, which was used for comparison with the *χ* ^2^ enrichment and McNemar tests.^37^ Statistical significance at the network level was determined using false discovery rate correction.

## RESULTS

### Study participants

Of 52 eligible children in the cohort with HIE, 40 children (77%) completed the fMRI scanning along with 43 in the control cohort. The MABC‐2 was assessed in all 40 children with successful fMRI in the cohort with HIE and in 24 children (56%) in the control cohort. Children who did not complete the fMRI or the MABC‐2 assessment were excluded from the brain–behaviour analysis. Eight children were further excluded from the analysis based on our criteria for excessive head movement (five from the cohort with HIE and three in the control cohort). The final study samples in our brain functional connectivity–motor performance association analysis included 35 children in the cohort with HIE and 21 children in the control cohort (a flow chart outlining this is shown in Figure S1). The basic characteristics of the cohort with HIE and the control cohort are presented in Table 1. Note that age at both fMRI and MABC‐2 differed significantly between the two groups (*p* < 0.001). A sensitivity analysis of the basic characteristics of both cohorts is presented in Table S1. Data pertaining to neonatal information in both cohorts, including an expanded sensitivity analysis regarding the included and excluded children in the cohort with HIE, have been presented previously.^12^ As already noticeable from the sensitivity analysis, 69% of children with grade II HIE (according to the Sarnat staging) were included in the final study sample but only 40% of children with grade III HIE were included. Recorded clinical data of the current diagnosis involving motor function detected one child with mild CP (classified in level I of the Gross Motor Function Classification System^41^) and one child with developmental coordination disorder (DCD) in the cohort with HIE at the time of the follow‐up, with a further four children having received an attention‐deficit/hyperactivity disorder (ADHD) diagnosis.

**TABLE 1:** Characteristics of the study population included in final analyses stratified according to exposure to neonatal HIE.

Characteristic	Group with HIE (*n* = 35)	Control group (*n* = 21)	*χ* ^2^	*p*
Female, *n* (%)	19 (54.3)	10 (47.6)	0.043	0.84^a^
Male, *n* (%)	16 (45.7)	11 (52.4)		

			*t*	*p*
Gestational age at birth (weeks), median (IQR)	40.6 (39.2–41.2)	40.1 (39.4–41.3)	0.48	0.63
Birthweight (g), median (IQR)	3500 (3326–3984)	3615 (3455–3745)	0.41	0.68
Age at MABC‐2 (years), median (IQR)	11.3 (10.5–12.8)	12.1 (11.9–12.2)	4.8	< 0.001
Age at fMRI (years), median (IQR)	11.4 (10.5–11.8)	10.5 (9.1–10.6)	4.9	< 0.001
CP diagnosis, *n* (%)	1 (2.9)	0 (0)	NA	NA
DCD diagnosis, *n* (%)	1 (2.9)	0 (0)	NA	NA
ADHD diagnosis, *n* (%)	4 (11.4)	0 (0)	NA	NA
Framewise displacement during fMRI acquisition (mm), mean (SD)	0.19 (0.13)	0.14 (0.07)	1.65	0.11
DVARS (standardized) during fMRI acquisition (mm), mean (SD)	1.14 (0.07)	1.16 (0.08)	1.44	0.16

Abbreviations: ADHD, attention‐deficit/hyperactivity disorder; CP, cerebral palsy; DCD, developmental coordination disorder; fMRI, functional magnetic resonance imaging; HIE, hypoxic‐ischaemic encephalopathy; IQR, interquartile range; MABC‐2, Movement Assessment Battery for Children, Second Edition; NA, not applicable.

^a^ *p*‐value derived using the *χ* ^2^ test; Yates correction was applied.

### Motor performance

**FIGURE 1:**
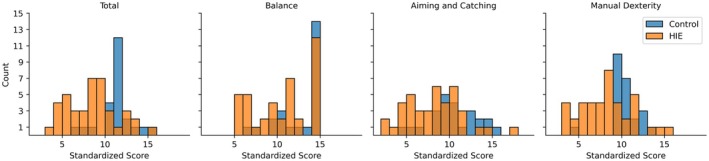
Distribution of the Movement Assessment Battery for Children, Second Edition standard scores for the cohort with hypoxic‐ischaemic encephalopathy (HIE) (*n* = 35) and the control (*n* = 24) cohort respectively.

The MABC‐2 standard score distributions were non‐normally distributed for the total score and for the manual dexterity and balance subdomains based on at least one cohort failing to achieve normality (Figure 1 and Table S2). Testing the difference between groups (i.e. the cohort with HIE and the control cohort) and subsequent correction for multiple comparisons across all MABC‐2 standard scores (four tests, corrected significance threshold, *p* < 0.013) detected significantly lower standard scores for the cohort with HIE in all MABC‐2 subdomains and total score (Table 2).

**TABLE 2:** Movement Assessment Battery for Children, Second Edition, stratified according to exposure to neonatal HIE treated with therapeutic hypothermia.

Measure	Group with HIE (*n* = 40)	Control group (*n* = 24)	*U*	*p*
Manual dexterity	7.5 (5.75–9)	10 (9–10.25)	240.5	< 0.001^a^
Balance	10 (6–14)	14 (10–14)	304.5	0.012^a^
Total	8 (5.75–9)	11 (10–11)	199	< 0.001^a^

			*t*	*p*
Aiming and catching	8 (5–10)	10 (8.75–12)	2.87	0.006^a^

Abbreviation: HIE, hypoxic‐ischaemic encephalopathy.

*Note*: Data are the standard score and interquartile range (IQR).

^a^ Significant after correction for multiple comparisons using Bonferroni correction (four tests, corrected significance threshold, *p* < 0.013). For each measure, the median standard score and IQR are presented with the test statistic and *p*‐value derived using a Mann–Whitney *U* test (for non‐normally distributed MABC‐2 scores) or *t*‐test (for normally distributed MABC‐2 scores).

### Functional brain connectivity at the level of individual brain regions

**FIGURE 2:**
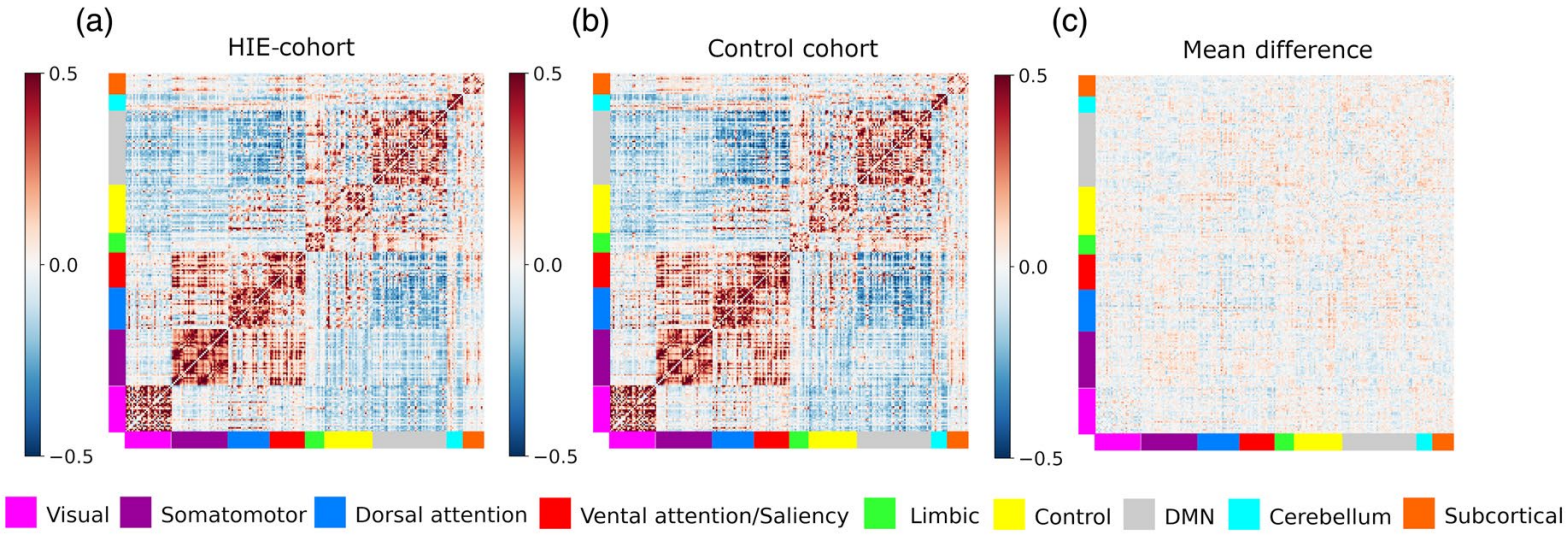
Mean resting‐state fMRI pairwise (region‐based) brain region connectivity matrices for the cohort with hypoxic‐ischaemic encephalopathy (HIE) (a) and the control cohort (b), and the mean difference in functional connectivity (c). Abbreviation: DMN, default mode network.

Based on the applied brain parcellation scheme, separate correlation matrices of pairwise brain region functional connectivity were computed in both cohorts and used for the subsequent brain network–motor score performance association analysis. Consistent with previous studies, both cohorts demonstrated strong within‐network and between‐network correlation and a mix of positive and negative correlations between networks, with smaller differences between cohorts (Figure 2).

### Assessment of the associations between brain connectivity and motor performance

**FIGURE 3:**
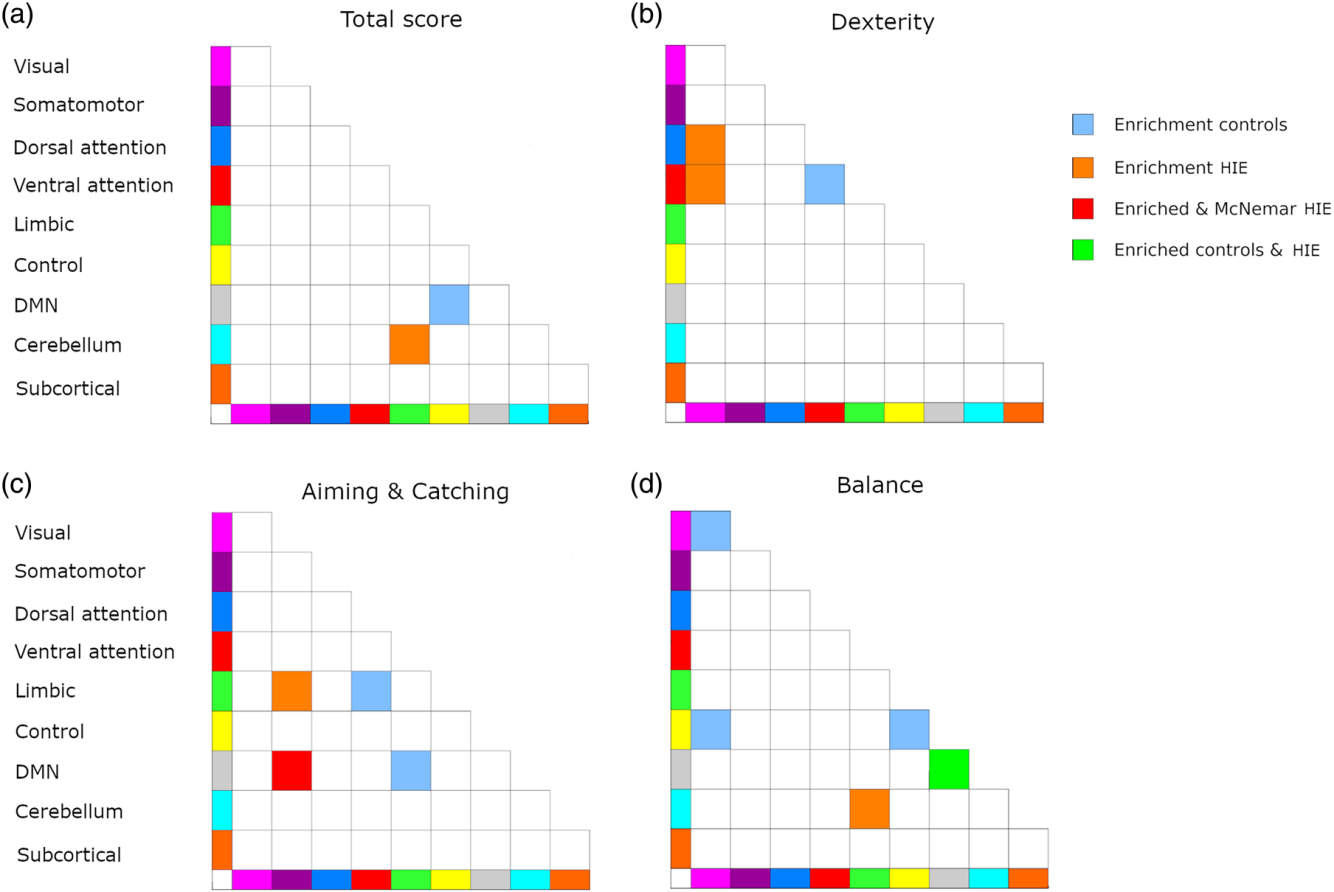
Brain subnetwork‐level enrichment of correlations between pairwise brain region functional connectivity measures and scores of motor performance according to the Movement Assessment Battery for Children, Second Edition. (a) Total score. (b) Dexterity. (c) Aiming and catching. (d) Balance. Abbreviations: DMN, default mode network; HIE, hypoxic‐ischaemic encephalopathy.

The results from the enrichment analysis of the associations between network‐level brain functional connectivity and MABC‐2 motor performance scores are shown in Figure 3. All MABC‐2 total and subdomain parameters of motor performance showed significantly enriched clusters of correlation between measures of paired brain region functional connectivity and motor scores in both cohorts.

#### Movement Assessment Battery for Children, Second Edition: Total score

Regarding the total MABC‐2 standard score in the control cohort, an enriched cluster of correlations between behaviour and pairwise brain functional connectivity was present between the default mode and control networks (Figure 3a). For the cohort with HIE, an enriched cluster of functional correlations was found between the cerebellar and limbic networks. No significant group differences were detected between brain functional connectivity and the total MABC‐2 standard score.

#### Movement Assessment Battery for Children, Second Edition: Manual dexterity subdomain

For the MABC‐2 manual dexterity subdomain (Figure 3b), significant clusters of enrichment for pairwise brain functional connectivity and behaviour were observed between the visual and dorsal, and the ventral attention/saliency, networks in the cohort with HIE. Moreover, within‐network pairwise brain region functional connectivity measures in the ventral attention/saliency network were enriched in the control cohort. As with the MABC‐2 total score subtest, no significant group differences in brain functional connectivity and behaviour relationships were detected for the manual dexterity subdomain.

#### Movement Assessment Battery for Children, Second Edition: Aiming and catching subdomain

**FIGURE 4:**
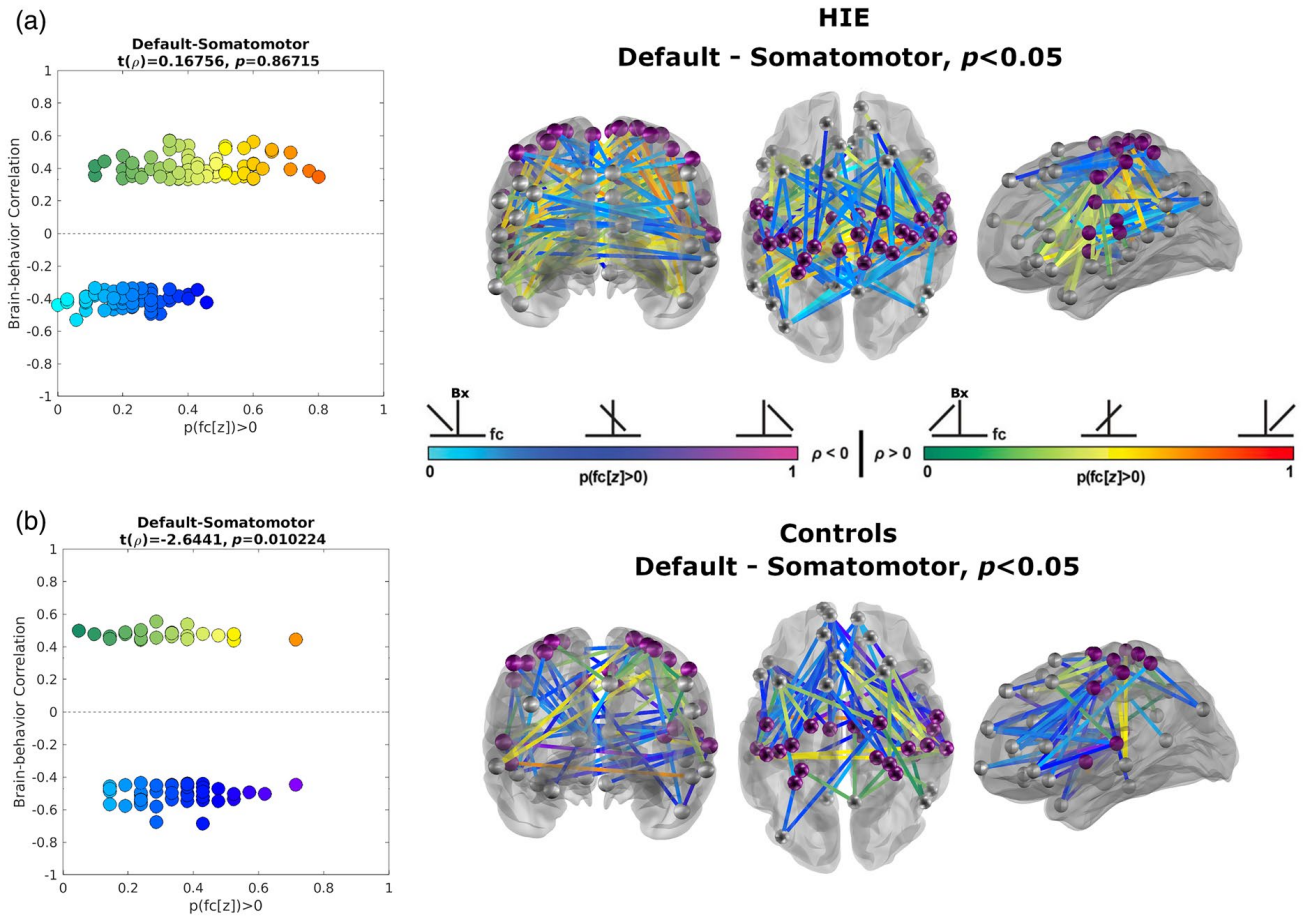
The degree of functional connectivity between the default mode network and the somatomotor network was significantly associated with the aiming and catching subdomain of the Movement Assessment Battery for Children, Second Edition (MABC‐2). Enrichment analysis identified significant clusters of region of interest (ROI)‐based functional connectivity associated with the total standard score of the MABC‐2 aiming and catching subdomain score for the cohort with hypoxic‐ischaemic encephalopathy (HIE) (a) but not the control group (b), with a significant difference between groups. The scatter plots present the enriched brain–behaviour correlation between individual ROI pairs of functional connectivity (default mode network and somatomotor network; see Figure 3c) and the MABC‐2 aiming and catching subdomain score. Light blue to magenta reflects ROI pairs with a negative correlation slope; green to red reflects a positive correlation slope. The anatomical localizations of the enriched ROI pairs are presented on glass brain template in the right column of (a) and (b), using the same colour maps.

A significant clustering of correlations between brain functional connectivity and the MABC‐2 aiming and catching subdomain (Figure 3c) was observed in the cohort with HIE between the limbic and default mode network (DMN) pair, between the somatomotor and limbic network pair, and between the somatomotor and DMN pair. In the latter, the degree of clustering was also significant at a group level, with the cohort with HIE exhibiting a larger proportion of significant functional connectivity pairs with both negative and positive brain–behaviour correlations, and both stronger negative and positive correlations, than the control cohort (Figure 4). Additionally, the size of the correlation coefficient was greater for both negative and positive brain–behaviour correlations compared to the controls. Notably, the larger number of enriched region of interest pairs in the cohort with HIE spanned intrahemispheric and interhemispheric brain connections.

For the control cohort, enriched clustering of the correlation between brain functional connectivity and the total standard score of the MABC‐2 aiming and catching subdomain was present for the limbic–ventral attention/saliency network pair.

#### Movement Assessment Battery for Children, Second Edition: Balance subdomain

Significantly enriched clusters of correlation were observed for the MABC‐2 balance subdomain (Figure 3d), both within and between the visual and control networks in the control cohort. Additionally, within‐network functional connectivity for the DMN was enriched in the control cohort. In the cohort with HIE, the cerebellum–limbic network pair and within‐network functional connectivity in the DMN were clustered and enriched with regard to the MABC‐2 balance subdomain parameters. No significant group differences in brain subnetwork functional connectivity and behaviour relationships were detected for the balance subdomain.

## DISCUSSION

In this study, we investigated the relationship between several motor and higher associative cortical functional connectivity networks and motor performance in young adolescents treated with therapeutic hypothermia for neonatal HIE. Children exposed to therapeutic hypothermia‐treated neonatal HIE obtained significantly (*p* < 0.05) lower scores compared to controls in the MABC‐2 assessment, for both the total score and in all three subdomains. Using statistical enrichment analysis, we found several significant clusters of correlation between measures of paired brain region functional connectivity and motor performance for both total and subdomain MABC‐2 scores in the cohort with HIE and in the control cohort. Interestingly, enriched functional connectivity between the cerebellum and limbic network (i.e. bilateral orbitofrontal cortex and bilateral temporal pole) correlated with motor performance for both the MABC‐2 total and balance subdomain scores in the cohort with HIE. This indicates a possible alternative route of neural processing related to motor function in children treated with therapeutic hypothermia for neonatal HIE. However, these results did not differ significantly compared to the control cohort. On the other hand, correlation with the MABC‐2 aiming and catching subdomain detected enriched default mode–somatomotor pair functional connectivity in the cohort with HIE, which also differed significantly compared to the control cohort. While the somatomotor network is central for motor function, the DMN decreases during activity in goal‐oriented tasks and increases during self‐referential processes and mind‐wandering.^42^, ^43^ The DMN is involved in motor function for processes such as motor learning^44^ and the preparation of movement (i.e. motor readiness).^45^ Our results show that the DMN–somatomotor network interaction also correlates with motor performance in children treated with therapeutic hypothermia for neonatal HIE. However, a significant DMN–somatomotor network connection was only found in one of the three dimensions tested in the MABC‐2 in our cohort (i.e. aiming and catching). Although these subdomains are designed to provide independent measures of different strands of motor development,^14^ we nevertheless consider that a more consistent finding in brain functional connectivity–motor performance across the entire MABC‐2 would have confirmed a neural basis for decreased motor and coordination ability after therapeutic hypothermia‐treated neonatal HIE.

In an evaluation of cognitive and motor function in a cohort of 27 children without CP aged 5 to 7 years treated with therapeutic hypothermia for neonatal HIE, Erdi‐Krausz et al.^4^ found a similar decreased motor performance as in our study, which was further associated with high scores of inattention. Therefore, they suggested that inattention may have contributed to the non‐CP motor impairment seen in this group. In support of this, the DMN has been shown to be important for attentional lapses with momentary self‐referential processes.^46^ However, the DMN has also been implicated in other processes, with possible implications for goal‐directed activity, such as error monitoring and self‐related evaluation,^47^ as well as voluntary decision processing.^48^ Therefore, it is plausible that the DMN and its role in error monitoring and self‐evaluation explains our finding of an association between motor performance and the DMN–somatomotor network functional connectivity. Importantly, cognitive function was assessed in this cohort of children treated with therapeutic hypothermia for neonatal HIE by Robertsson Grossmann et al.;^13^ they found it to be normal (Full‐scale IQ = 101.5 assessed with the Wechsler Intelligence Scale for Children, Fifth Edition). To further investigate the possible impact of cognitive ability on our findings, we tested for a correlation between individual MABC‐2 scores and Full‐scale IQ (using Pearson's rank correlation coefficients for normally distributed scores and Spearman's rank correlation coefficients for non‐normal distributions) but found no significant correlation after subsequent Bonferroni correction of *p*‐values for multiple comparisons (four two‐sided tests, *p* = 0.006) (Table S3). Therefore, we think it is unlikely for cognitive capacity or general development per se to be the cause of the study findings, with enriched functional connectivity between the somatomotor network and higher associative cortical networks.

Children exposed to neonatal HIE are at high risk for neurodevelopmental disorders such as ADHD and DCD.^13^, ^49^, ^50^ In ADHD, attention deficits are one of the main symptoms along with hyperactivity and impulsivity;^51^ increased activity in the DMN has been hypothesized to have a key role in impaired sustained attention.^52^ DCD, on the other hand, is a disorder affecting motor function caused by a dysfunctional interplay of motor and cognitive abilities, with impaired automatization and correction of movements in real time.^53^, ^54^ In a study of functional connectivity in children with DCD aged 8 to 12 years (*n* = 35), Rinat et al.^55^ detected significant changes between the somatomotor network and the posterior cingulate cortex and precuneus compared to typically developing controls (*n* = 23), both of which are integrated parts of the DMN.^56^ Therefore, it is also possible that our results reflect an increased degree of symptoms of these conditions in our cohort.

Wheelock et al.^38^ used the same modelling and statistical significance testing approach as presented by us in this article to investigate cortical network functional connectivity related to motor function in 58 children born very preterm. In agreement with the results presented in this article, these children born very preterm also obtained significantly lower MABC‐2 scores compared to 65 controls born at term. Moreover, Wheelock et al.^38^ found that children born very preterm had less enriched subcortical–cortical network pairs but increased enriched pairs both within and between networks, mostly involving higher associative cortical networks, including the DMN. These results align with the results from our study that motor function within the normal range is characterized by less recruitment of higher associative functional networks. The similarities between the study by Wheelock et al.^38^ and the results of our study indicate that the pattern of increased recruitment of higher‐order associative networks during the performance of motor tasks is not specific to the injury pattern of neonatal HIE (nor to treatment with therapeutic hypothermia). Rather, this pattern could constitute an adaptation for the brain to manage or compensate for some of the motor symptoms seen in these conditions. Alternatively, affected cognitive abilities, such as executive functions, which are impaired at the group level after both preterm birth^57^ and neonatal HIE,^50^ and the subsequent aberrant functional connectivity, could be the cause. Whether such abilities and associated functional connectivity networks correlate with the motor impairment seen in affected children should be investigated in future studies.

### Limitations

Several limitations need to be considered with regard to the interpretation of our results. First, the limited study sample restricts the power of the analysis. Although this is a common problem for studies investigating rare diagnoses, this nevertheless must be considered when assessing statistical robustness and sensitivity, and the ability to detect small changes in brain functional connectivity potentially associated with motor performance. Second, the study sample only included 35 (60%) of the surviving 58 children from the original cohort (see Figure S1 for the flow chart), and only 1 of the 10 surviving children who had developed CP had a mild‐enough impairment to complete testing and fMRI. Therefore, our results should only be generalized to children with therapeutic hypothermia‐treated neonatal HIE without CP and to Sarnat stage II HIE. Another prevalent problem to be considered is the increased risk of head‐motion‐related artefacts in the resting‐state fMRI data collected at a young age, in particular for children with ADHD and DCD.^58^ However, absence of a statistical group difference for the two measures of head movement used in this study (framewise displacement and DVARS) (Table 1) makes this source of bias less likely. Moreover, we minimized the influence of head movement bias by applying head‐motion exclusion criteria and denoising strategies that fulfil the proposed standards.^30^, ^32^

The choice of cortical parcellation scheme could also be a source for caution. We used a parcellation atlas based on data recorded in adults because of a lack of matching paediatric alternatives. Although resting‐state functional connectivity networks are stable in their spatial organization at early adolescence, brain volumes are smaller, thus putting higher demands on data‐driven image co‐registration pipelines. Additionally, there was a significant difference in age at scanning, and age at assessment with the MABC‐2, between the two groups. In our previous whole‐brain study of functional connectivity in children of this cohort,^12^ a slightly higher mean difference in age between groups compared to the cohorts in the current study resulted in noticeable effects on functional connectivity. Although the mean age difference at scanning in this study was smaller, it may still introduce bias in our analysis. Regarding the mean age difference at the MABC‐2 assessment, we used standardized scores adjusted for age, which have shown strong validity across ages, especially in older children.^14^

## Conclusions

Children treated with therapeutic hypothermia for neonatal HIE developed an elevated level of functional connectivity between specialized motor networks and higher associative cortical networks for motor performance. This suggests a possible neural basis contributing to the observed motor impairments in children with HIE.

## Supporting information


**Figure S1:** Flow chart of children treated with therapeutic hypothermia for HIE

**Table S1:** Sensitivity analysis for surviving children without genetic syndrome exposed to hypothermia‐treated neonatal HIE

**Table S2:** Shapiro–Wilk test for normality of scores from the MABC‐2

**Table S3:** Test for correlation of scores from the MABC‐2 with Full‐scale IQ from testing with Wechsler Intelligence Test for Children, Fifth Edition

## Data Availability

The datasets generated during and/or analyzed during the current study are available from the corresponding author upon reasonable request.
