# Supplementary figures and images for: Motor performance and higher associative cortical networks in adolescents with neonatal hypoxic‐ischaemic encephalopathy treated with therapeutic hypothermia
Source: Dev Med Child Neurol. 2025 Jun 22;68(1):99–109. doi: 10.1111/dmcn.16371 (PMC12683310; doi:10.1111/dmcn.16371)

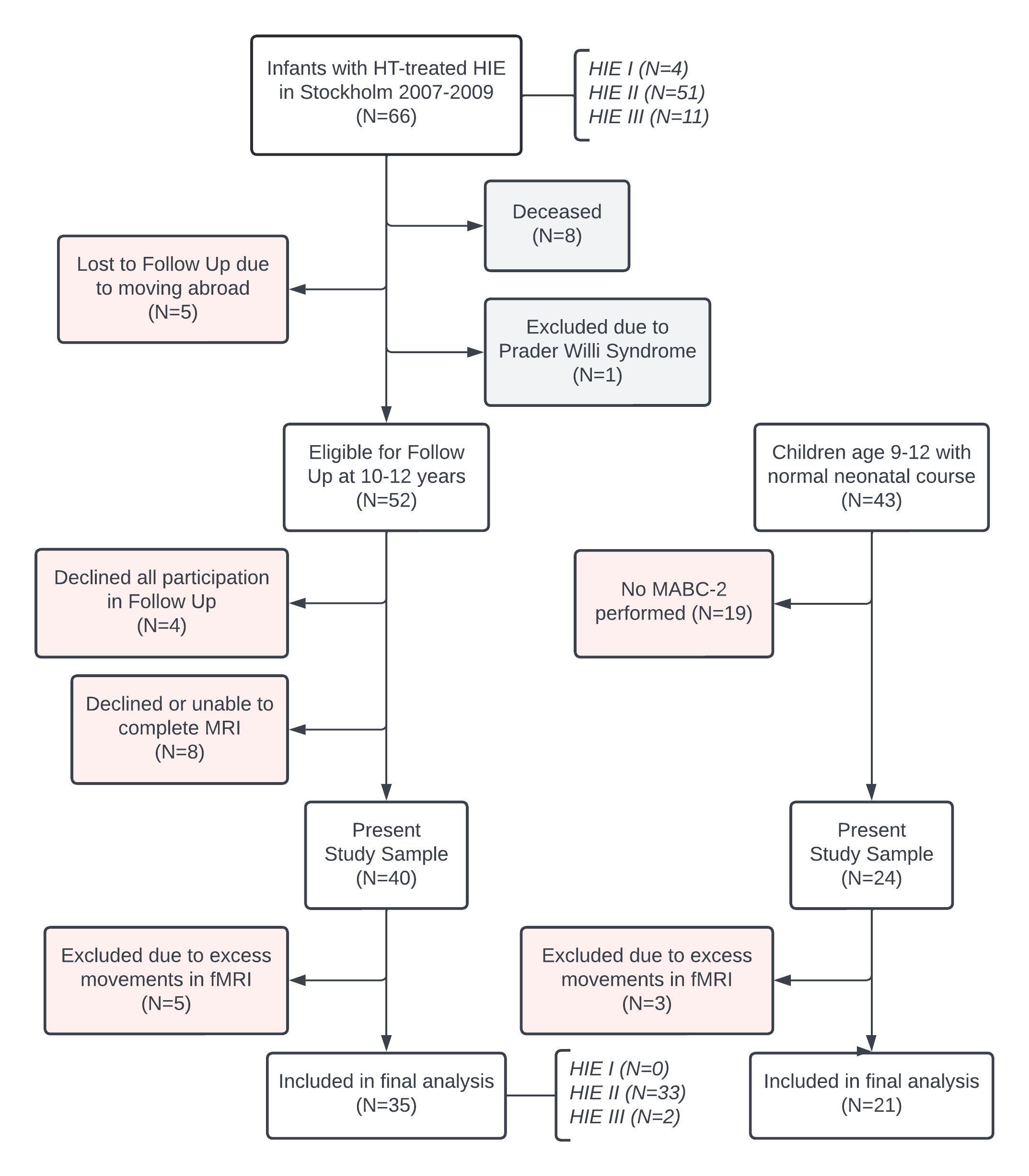

Supplement: Supplementary file 1 — Figure S1: Flow chart of children treated with therapeutic hypothermia for HIE [file DMCN-68-99-s004.jpeg]
