# Supplementary material for: Motor performance and higher associative cortical networks in adolescents with neonatal hypoxic‐ischaemic encephalopathy treated with therapeutic hypothermia
Source: Dev Med Child Neurol. 2025 Jun 22;68(1):99–109. doi: 10.1111/dmcn.16371 (PMC12683310; doi:10.1111/dmcn.16371)
Supplement: Supplementary file 2 — Table S1: Sensitivity analysis for surviving children without genetic syndrome exposed to hypothermia‐treated neonatal HIE [file DMCN-68-99-s003.docx]

**Supplemental Table 1** Sensitivity analysis for surviving children without genetic syndrome exposed to hypothermia-treated neonatal hypoxic-ischemic encephalopathy (n=57) and healthy controls with normal neonatal period (n=44), stratified by included or excluded from functional brain connectivity analysis.

| **Charectaristic** | **HIE included in analysis (n=35)** | **HIE excluded from analysis (n=22)** | **P** |
| --- | --- | --- | --- |
| Female sex, No (%) | 19 (54.3) | 11 (50.0) | 0.75 |
| GA, median (IQR), wk | 40.6 (39.2 - 41.2) | 40.5 (39.1 - 41.4) | 0.75 |
| BW, median (IQR), g | 3500 (3326 - 3984) | 3594 (3143 - 4340) | 0.52 |
| Apgar score at 10 minutes,  median (IQR) | 4 (2.5 - 6) | 5 (4 - 6) | 0.34 |
| *Sarnat grade of HIE* |  |  |  |
| Grade I, No (%) | 0 (0) | 4 (18.2) | 0.015 |
| Grade II, No (%) | 33 (94.3) | 15 (68.2) |  |
| Grade III, No (%) | 2 (5.7) | 3 (13.6) |  |
| WISC IV, full scale IQ at 6 to 8 years,  median (IQR) | 102.5 (97 - 109) | 107.5 (104 - 111)^a^ | 0.35 |
| Any Neurologic/Neurodevelopmental  diagnose and/or IQ < 85, No (%) | 14 (40) | 7 (31.8)^b^ | 0.61 |
|  | **Controls included in analysis (n=21)** | **Controls excluded from analysis (n=23)** | **P** |
| Female sex, No (%) | 11 (52.4) | 11 (47.8) | 0.76 |
| GA, median (IQR), wk | 40.0 (39.3 – 41.2) | 39.9 (39.2 – 40.7) | 0.54 |
| BW, median (IQR), g | 3615 (3455 – 3745) | 3710 (3329 – 3899) | 0.77 |

Abbreviations: BW, Birth Weight; GA, Gestational Age; HIE, Hypoxic-Ischemic Encephalopathy; IQ, Intelligence Quote; IQR, Intra Quartile Range; WISC, Wechsler Intelligence Scale in Children;

^a^Available for 12 (55%) of the children

^b^At age 6-8 years
