# Supplementary material for: Motor performance and higher associative cortical networks in adolescents with neonatal hypoxic‐ischaemic encephalopathy treated with therapeutic hypothermia
Source: Dev Med Child Neurol. 2025 Jun 22;68(1):99–109. doi: 10.1111/dmcn.16371 (PMC12683310; doi:10.1111/dmcn.16371)
Supplement: Supplementary file 3 — Table S2: Shapiro–Wilk test for normality of scores from the MABC‐2 [file DMCN-68-99-s001.docx]

**Supplemental Table 2.** Results from Shapiro-Wilks test for normality of scores from Movement Assessment Battery for Children 2^nd^ ed. stratified by exposure to neonatal hypoxic-ischemic encephalopathy treated with therapeutic hypothermia.

| Measure | HIE cohort (n=35) | | Control cohort (n=21) | |
| --- | --- | --- | --- | --- |
|  | **W** | **P** | **W** | **P** |
| Manual Dexterity | 0.96 | 0.23 | 0.86 | 0.0026* |
| Balance | 0.97 | <0.001 | 0.76 | <0.001* |
| Aiming and Catching | 0.97 | 0.29 | 0.97 | 0.60 |
| Total | 0.96 | 0.24 | 0.89 | 0.013* |

Abbreviations: HIE, Hypoxic-Ischemic Encephalopathy

*Non-normal distribution based on testing with the Shapiro-Wilks test
