# Supplementary material for: Motor performance and higher associative cortical networks in adolescents with neonatal hypoxic‐ischaemic encephalopathy treated with therapeutic hypothermia
Source: Dev Med Child Neurol. 2025 Jun 22;68(1):99–109. doi: 10.1111/dmcn.16371 (PMC12683310; doi:10.1111/dmcn.16371)
Supplement: Supplementary file 4 — Table S3: Test for correlation of scores from the MABC‐2 with Full‐scale IQ from testing with Wechsler Intelligence Test for Children, Fifth Edition [file DMCN-68-99-s002.docx]

**Supplemental Table 3.** Results from test for correlation of scores from Movement Assessment Battery for Children 2^nd^ ed. with full scale IQ from testing with Wechsler Intelligence Test for Children, 5^th^ version, stratified by exposure to neonatal hypoxic-ischemic encephalopathy treated with therapeutic hypothermia.

| Measure | HIE cohort (n=35) | | Control cohort (n=21) | |
| --- | --- | --- | --- | --- |
|  | **ρ** | **P** | **ρ** | **P** |
| Dexterity | 0.28 | 0.11 | 0.24 | 0.30 |
| Balance | 0.04 | 0.83 | 0.51 | 0.019 |
| Total | 0.17 | 0.34 | 0.12 | 0.61 |
|  | **r** | **P** | **r** | **P** |
| Aiming and Catching | 0.08 | 0.67 | -0.23 | 0.31 |

Abbreviations: HIE, Hypoxic-Ischemic Encephalopathy; IQ, Intelligence Quotient
